# Hydroxyethyl Starch Curcumin Enhances Antiproliferative Effect of Curcumin Against HepG2 Cells *via* Apoptosis and Autophagy Induction

**DOI:** 10.3389/fphar.2021.755054

**Published:** 2021-11-03

**Authors:** Linhai Jiang, Xiaoliang Cai, Sheng Li, Ying Miao, Xiaoyu Yang, Manbian Lin, Liang Chen, Xiaozheng He, Zuquan Weng

**Affiliations:** ^1^ Fuzhou Traditional Chinese Medicine Hospital, Fuzhou, China; ^2^ College of Biological Science and Engineering, Fuzhou University, Fuzhou, China

**Keywords:** hydroxyethyl starch, curcumin, hepatocellular carcinoma, apoptosis, autophagy, mitochondria

## Abstract

It is well documented that curcumin (CUR), as a polyphenol molecule originated from turmeric, has many advantages such as antioxidative, anti-inflammatory, neuroprotective, and antitumor effects. However, because of its poor water solubility and low bioavailability, the biomedical applications of CUR are limited. So, in this study, we modified CUR with conjugation to a food-derived hydrophilic hydroxyethyl starch (HES) *via* an ester linkage to fabricate the amphiphilic conjugate HES-CUR prior to self-assembling into uniform nanoparticles (HES-CUR NPs). And, the results of the ^1^H NMR spectra and FT-IR spectrum showed successful synthesis of HES-CUR NPs; moreover, the solubility and the drug loading efficiency of CUR were significantly increased. Next, we further explored the differences on the antitumor effects between HES-CUR NPs and CUR in HepG2 cells, and the results of the CCK8-assay and cell counting experiment showed that HES-CUR NPs exhibited a more significant antiproliferative effect than that of CUR in HepG2 cells. And HepG2 cells were more sensitive to apoptosis induced by HES-CUR NPs as evidenced by flow cytometry, increased cytochrome c level, and decreased full length caspase-3 and Bcl-2 protein expressions. Additionally, we found that the efficacy of HES-CUR NPs against HepG2 cells might be related to the enhanced degree of mitochondrial damage (decrease of the mitochondrial membrane potential and ATP) and autophagy (increased levels of Beclin-1 and LC3-II proteins). So, the findings in this study suggest that HES-CUR NPs have a great application potential in antitumor efficacy and play an important role in multiple signal pathways.

## Introduction

Hepatic carcinoma, as the most common primary malignant tumor, is characterized by a high incidence in the most populous areas of the world, accounting for about 90% of all primary liver cancers (Petruzziello 2018). More than 700,000 new cases are diagnosed each year, and 600,000 deaths occur worldwide each year, accounting for 9.2% of all new cancer cases all around the globe (Chedid et al., 2017; Grandhi et al., 2016; Jemal et al., 2011; Jiang et al., 2017; Petruzziello 2018). In recent years, aiming to help patients with hepatocellular carcinoma to a certain degree, researchers have continuously focused their attention to natural biologically active molecules. Among the substantial natural active molecules, curcumin (CUR) becomes a hotspot on account of its blood lipid–lowering, antitumor, anti-inflammatory, and antioxidant potential. As a diketone compound extracted from the rhizomes of Zingiberaceae plants, CUR can provide some benefits for patients with chronic diseases (Kotha and Luthria 2019; Lestari and Indrayanto 2014; Menon and Sudheer 2007; Nelson et al., 2017). Many studies showed that CUR treatment has an inhibitory effect on the proliferation of HepG2 cells (Goel et al., 2008; Gupta et al., 2013).

Meanwhile, the mechanism studies on CUR related to anticancer effects were reported. Muangnoi H et al. verified that CUR inhibited the proliferation process of HepG2 cells by promoting apoptosis (Muangnoi et al., 2019). Mitochondria play a pivotal role in the apoptotic process. A decrease in mitochondrial transmembrane potential was the early signal stimulus that caused the apoptotic cascade response, and apoptosis resulted from the disruption of mitochondrial transmembrane potential was irreversible (Kocyigit and Guler 2017). Altered mitochondrial membrane permeability induced the upregulation of the Bcl-2 family of proapoptotic proteins, Bax, and downregulation of the antiapoptotic protein, Bcl-2, and prompted the release of cytochrome c from the mitochondria into the cytoplasm, leading to the activation of the caspase family of apoptotic execution protein, caspase-3, and ultimately triggering the apoptotic process in cells (Maiuri et al., 2007; Pistritto et al., 2016). On the other side, autophagy, an integral biological process critical to cellular and organismal health, is defined as an evolutionarily conserved cyclic process in which intracellular macromolecules are broken down into their component parts within the lysosomes (Levy et al., 2017). Previous studies have demonstrated that CUR affected intracellular autophagic function by targeting various stages of the autophagic signaling molecules, including LC3-II, cytochrome c, and Beclin-1 (Glick et al., 2010; Onorati et al., 2018).

The studies mentioned above further suggested that CUR played an important role in human health care. However, the low bioavailability of CUR due to its poor property of aqueous solubility reduced the actual effectiveness of treatment and prevention in diseases (Nelson, Dahlin, Bisson, Graham, Pauli and Walters 2017). In recent years, researchers tried to use some common methods to address these issues mainly involved in various colloidal nanoparticles, including polymers, lipids, and nanoparticles (Anand et al., 2007; Araújo and Leon 2001), but failed to avoid the defects of binding proteins randomly and increasing water solubility to a certain limit. It is well accepted that hydroxyethyl starch (HES) is a semi-synthetic degradable polymer, which is widely employed as a plasma volume–increasing agent (Haase and Perner 2013; Joosten et al., 2020). HES also has controllable biodegradation features attributed to the fact that it could often be easily degraded by α-amylase in serum *in vivo* (De Hert and De Baerdemaeker 2014; Myburgh et al., 2013). Additionally, it has high water solubility, low sensitivity, and no protein rejection characteristics. So, as an essential biomedical material, HES has been proven to extend the circulation time of drugs with a short half-life (Perner et al., 2012). Owing to these merits, HES was employed in this study to modify CUR by means of reacting with monocarboxylic curcumin (CUR-COOH) to construct the hydroxyethyl starch curcumin (HES-CUR) polymer via the esterification reaction. We postulate that the advantages of HES can be utilized to improve the low water solubility of CUR, so as to achieve its favorable bioavailability as well as stability.

In this study, we prepared HES-CUR NPs by combining CUR with HES to increase the aqueous solubility of CUR and the drug loading efficiency of CUR. After this, we studied the differences between CUR and HES-CUR NPs, including antiproliferative effects and their possible mechanisms in HepG2 cells.

## Materials and Methods

### Synthesis of Hydroxyethyl Starch Curcumin Conjugates

The method of monocarboxylic-terminated CUR (CUR-COOH) generation was described in the previously published article (Xu et al., 2018; Chen et al., 2020) with slight modifications. First, we made an adjustment of the feed ratio of ingredients: CUR (13.26 g) was dissolved in pyridine (200 ml) and glutaric anhydride acid (4.24 g) was dissolved in pyridine (76 ml). The crude product CUR-COOH was obtained through extraction and vacuum-drying prior to purification by silica gel column chromatography. Subsequently, the CUR-COOH obtained was used to prepare the conjugates of HES-CUR.

### Preparation of Hydroxyethyl Starch Curcumin Nanoparticles

HES-CUR NPs were prepared by the self-assembly of HES-CUR conjugates in the manner of nanoprecipitation. In brief, a HES-CUR polymer (5 mg) was dissolved in 1 ml DMSO, and the solution was slowly injected into 2 ml ultrapure water using a syringe pump prior to stirring within 1 h. Subsequently, the mixture was dialyzed using ultrapure water for 24 h (MWCO: 3,500 Da) continuously. The dialyzed sample was lyophilized to obtain HES-CUR NPs as yellow powders. Then, size distribution and zeta potential of the nanoparticles were analyzed using a zetasizer (Brookhaben, America) and transmission electron microscope (TEM) (FEI TECNAI G2 F20, America).

Apart from recording the nuclear magnetic resonance (^1^H NMR) spectra of CUR-OH and HES-CUR NP conjugates on a Bruker 400 MHz spectrometer when deuterated DMSO was employed as a solvent, the structures of HES, CUR, and HES-CUR NPs were studied by using infrared spectroscopy (FT-IR) (Thermo Nicolet, America).

### Drug Loading Efficiency of Hydroxyethyl Starch Curcumin Nanoparticles

For the sake of determining the drug loading efficiency [DLE (%)] of HES-CUR NPs, a certain amount of lyophilized HES-CUR NPs was weighed and dissolved into a certain volume of a mixture of HCl solution and DMSO (v/v = 1/2.5), and the solution was subjected to an ultrasound at 37°C for 24 h until CUR was completely released. After the solution of CUR was filtered, the CUR concentrations were detected by high-performance liquid chromatography (HPLC, C18 column: 4.6 mm 250 mm, 5 m) at a wavelength of 425 nm. A mixture of acetonitrile and ultrapure water (7:3, v/v) was employed as the mobile phase, and the flow rate was 1.00 ml/min. The DLE (%) is calculated according to the following formula:

$$\text{DLE }\left(\text{\%}\right)=\frac{{\text{C}}_{\text{CUR}}\text{V}}{{\text{M}}_{\text{HES}-\text{CUR NPs }}}\times 100\text{\%}$$

in which C_CUR_ is the concentration of released CUR, V is the volume of release medium, and M_HES-CUR NPs_ is the total mass of CUR in the HES-CUR NPs.

### Toxic Test of Curcumin and Hydroxyethyl Starch Curcumin Nanoparticles in 3T3 Cells

The normal 3T3 cells (mouse embryonic fibroblasts) were used to analyze the toxic effects of CUR and HES-CUR NPs. The cells were seeded in a 96-well plate at a density of 1 × 10^4^ cells/well and cultured in a humid atmosphere of 37°C, 5% CO_2_ for 24 h. The old medium was replaced with 200 μL of the fresh medium per well, and then CUR and HES-CUR NPs were added to the culture medium at final concentrations of CUR 10–100 μg/ml (CUR DLE (%) of HES-CUR NPs was equal to that of CUR). After incubating for 48 h, 100 μL of the fresh medium and 10 μl of CCK-8 solution were added to each well and then incubated for 2 h. The absorbance of each well was measured using a microplate reader at 450 nm (Bio Tek, Synergy H1, United States). The relative cell viability was calculated by comparing the absorbance of the treatment sample and that of the corresponding control. Moreover, trypan blue exclusion test was performed to measure cell viability using a hemocytometer.

### Measurement of Proliferation Status in HepG2 Cells Treated by Curcumin and Hydroxyethyl Starch Curcumin Nanoparticles

HepG2 cells were cultured in Dulbecco’s modified Eagle medium (DMEM). The treatment concentrations of CUR and HES-CUR NPs, and the two methods used for measurement of their antiproliferative effects on HepG2 cells, were the same as those of 3T3 cells mentioned above.

### *In Vitro* Cell Uptake Assay

HepG2 cells were incubated in a glass-bottomed dish for 24 h and subsequently treated with HES-CUR NPs (equivalent to 125.00 μg/ml CUR) for 4 h. After that, the cells were washed thrice with PBS (2 ml, pH 7.4) and then fixed with paraformaldehyde for 10 min. Next, the cells were re-washed thrice with PBS and treated with DAPI (1.00 μg/ml) for 15 min to label the nuclei. Finally, the cells rinsed with PBS were mounted on a petridish for observation using a confocal laser scanning microscope (CLSM, A1, Nikon) using FITC filters (excitation at 488 nm) to monitor the localization of CUR in HepG2 cells.

In addition, HepG2 cells were treated with ultra-high concentrations (375.00 μg/ml) of CUR, and CLSM was used to observe the intracellular fluorescence intensity. Also, we measured the intracellular content of CUR by HPLC as described in the Methods section 3.

### Measurement of Mitochondrial Membrane Potential

MMP was measured following the procedures of the JC-10 kit produced by Sigma (St. Louis, MO, United States). Briefly, HepG2 cells were seeded onto 96-well plates at a density of 2 × 10^4^ cells per well. After 24 h, the cells were treated with CUR and HES-CUR NPs at final concentrations of10–100 μg/ml CUR for 24 h. 30 μl JC-10 dye was then added to each well and incubated for 30 min. Finally, the fluorescence intensity was detected using the chemiluminescent channel at 490/525 nm and 540/590 nm, respectively. And, the change in MMP was determined by calculating the relative ratio of the fluorescence intensity between 490/525 nm and 540/590 nm.

### Flow Cytometric Analysis

The HepG2 cells for apoptosis were examined using 1 × Annexin V binding solution (4A Biotech, Shanghai, China). HepG2 cells were seeded at a density of 2 × 10^5^ cells/well in 6-well plates. The HepG2 cells were treated with 100 μg/ml DLE (%) of CUR for 24 h. After treatment, the cells were washed with cold PBS. The cell pellets were then incubated in 100 µl annexin V binding buffer containing 5 µl of annexin V for 5 min and were then resuspended with propidium iodide (PI) in dark at 4°C for 5 min. The fluorescence intensity representing the apoptotic cells was analyzed using a BD *Accuri*™ C6 Plus flow cytometer (BD Biosciences, United States) using annexin V/Alexa Fluor 647 (with the excitation wavelength at 651 nm and emission wavelength at 667 nm) and PI (with the excitation wavelength at 535 nm and emission wavelength at 615 nm). The flow cytometry data were analyzed using FlowJo software.

### Western Blot Analysis

HepG2 cells treated with CUR and HES-CUR NPs were lysed using an ice-cold lysis buffer. The lysates were centrifuged at 13,500 g for 5 min at 4°C, and then the supernatant was used for the analysis of Western blot. Caspase-3 (Cell Signaling Technology, Beverly, MA; Catalog Number 9668), Bax (Cell Signaling Technology, Beverly, MA; Catalog Number 5023), Bcl-2 (Cell Signaling Technology, Beverly, MA; Catalog Number 4223), and cytochrome c (Cell Signaling Technology, Beverly, MA; Catalog Number11940) antibodies were used to determine apoptosis signals. LC3-II (Cell Signaling Technology, Beverly, MA; Catalog Number12741) and Beclin-1 (Cell Signaling Technology, Beverly, MA; Catalog Number4112) antibodies were used to determine autophagy signals. ImageLab software (ChemiDoc^TM^XRS+, Bio-Rad, America) was used to quantify the densities of the target bands. The results are expressed as the relative ratio of the band intensities of the target proteins and α-tubulin (Cell Signaling Technology, Beverly, MA; Catalog Number 3873) or β-actin (Cell Signaling Technology, Beverly, MA; Catalog Number 3700).

### Measurement of ATP

Collected cells from the 6-well plate after CUR treatment for 24 h were lysed, and cell lysates were centrifuged at 12,000 g at 4°C for 5 min. The supernatants were used as the sample to be tested. The levels of ATP in the cells were tested using the commercial kit produced by Beyotime Biotechnology Research Institute (Shanghai, China).

### Statistical Analysis

Multiple comparisons between the control and dose groups were performed using one-way analysis of variance, following by Dunnett’s post hoc test. Comparisons of two independent samples were performed using Student’s t-test. Data were presented as mean ± standard deviation (SD), and statistical analysis of the experimental data was calculated using OriginPro Software version 9.2.

## Results

### Structure Characterization and Drug Loading Determination of Hydroxyethyl Starch Curcumin Nanoparticles Polymer

**FIGURE 1:**
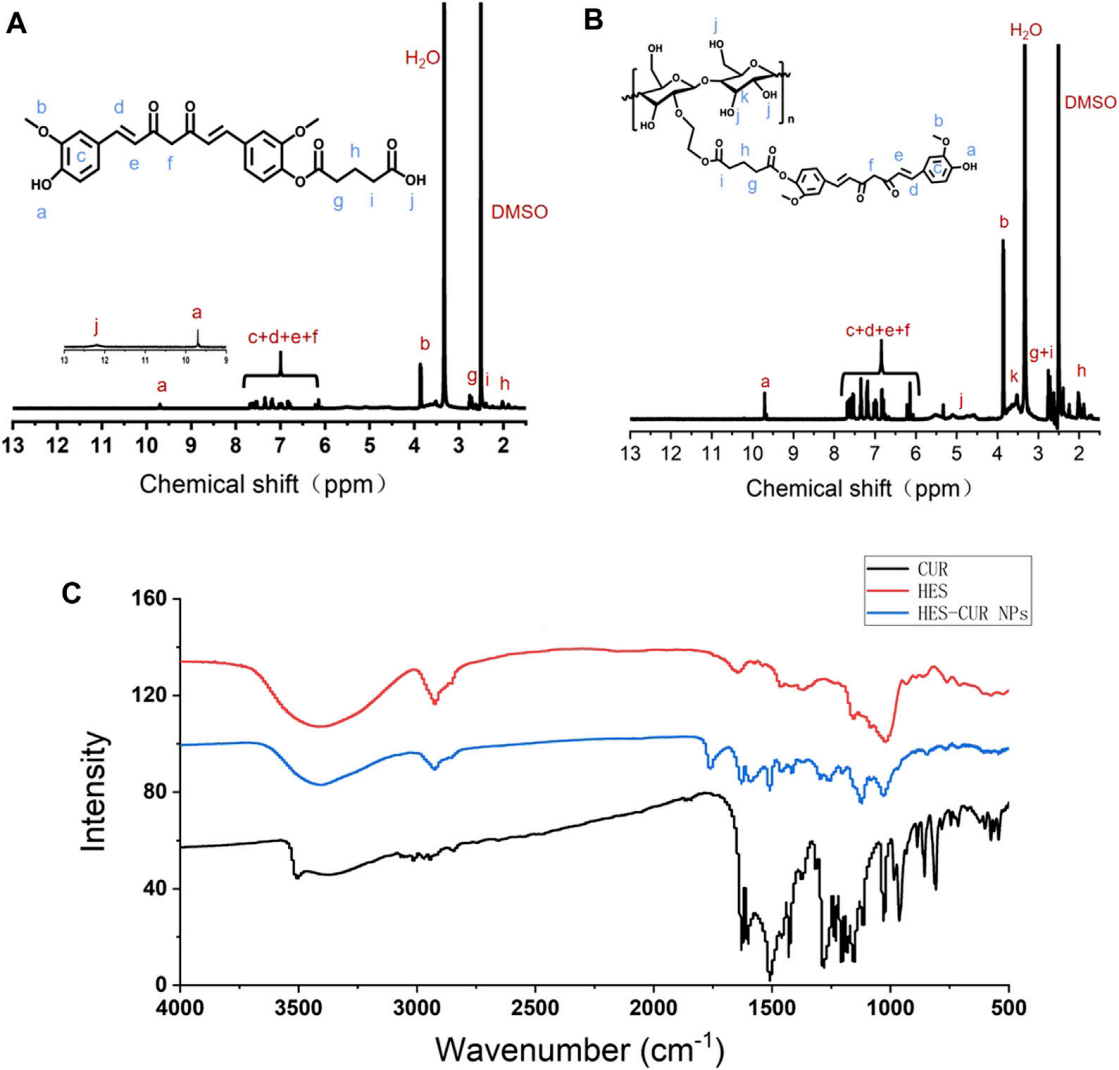
^1^H NMR spectra of **(A)** CUR-COOH and **(B)** HES-CUR NP conjugates, and **(C)** FT-IR spectrum of CUR, HES, and HES-CUR NPs.

The ^1^H NMR spectra verified that CUR was successfully aggregated with hydroxyl-containing HES to fabricate the HES-CUR NP conjugates by glutaric anhydride acid facilitating the esterification reaction. Figures 1A,B showed the peaks of the carbonyl (12.17 ppm) and methylene groups (1.86, 2.38, and 2.63 ppm), and suggested the successful yielding of CUR-COOH. Moreover, the appearance of the peaks representing CUR (9.73 ppm, 6.07–7.68 ppm, 3.85 ppm) indicated that the other part of CUR was complete.

**FIGURE 2:**
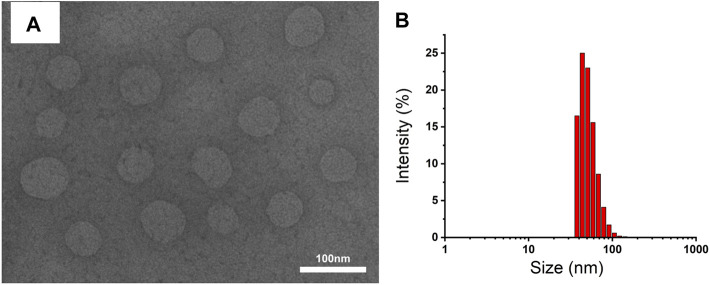
**(A)** TEM image and **(B)** size distribution of HES-CUR NPs.

The FT-IR assay was used to further confirm whether hydroxyl-containing HES was successfully incorporated into CUR-COOH. As exhibited in Figure 1C, when HES was aggregated to CUR-COOH, the peak location and shape of HES-CUR NPs were similar to that of HES at 1,159 cm^−1^ and 1,021 cm^−1^ (C–O–C stretching vibrations) and 2,927 cm^−1^ (the -CH_2_ stretching vibration) and 3,423 cm^−1^ (O–H stretching vibration), respectively. Meanwhile, signature peaks appeared at 1,628 cm^−1^ (aromatic moiety C=C stretching), 1,597 cm^−1^ (benzene ring stretching vibrations), and 1,509 cm^−1^ (C=O and C=C vibrations) in both profiles of HES-CUR NPs and CUR, indicating that CUR existed in the conjugates of HES-CUR. Moreover, images of HES-CUR NPs obtained from TEM demonstrated micellar nanoparticles with slightly irregular shapes (Figure 2A). Furthermore, the hydrodynamic diameter of HES-CUR NPs was determined by DLS (Figure 2B), and the average size was 71.97 nm with a PDI of 0.09, basically in line with that observed by TEM.

To explore the maximum concentration of HES-CUR NPs in water, we calculated the DLE (%) of CUR. In this study, the DLE (%) of CUR was significantly higher than that of our previous study (Chen et al., 2020), and the mean value was increased by nearly two fold (Table 1).

**TABLE 1:** Comparison of CUR loading efficacy of HES-CUR NPs between the current study and our previous study (Chen et al., 2020).

	Previous study	Current study
DLE (%)	13.28 ± 2.57	25.61 ± 4.09 *

Note: Data were expressed as mean ± SD, and the experiment was repeated thrice. *p < 0.05, compared to the previous study.

### Evaluation of Antiproliferative Effect of Curcumin and Hydroxyethyl Starch Curcumin Nanoparticles in HepG2 Cells

**FIGURE 3:**
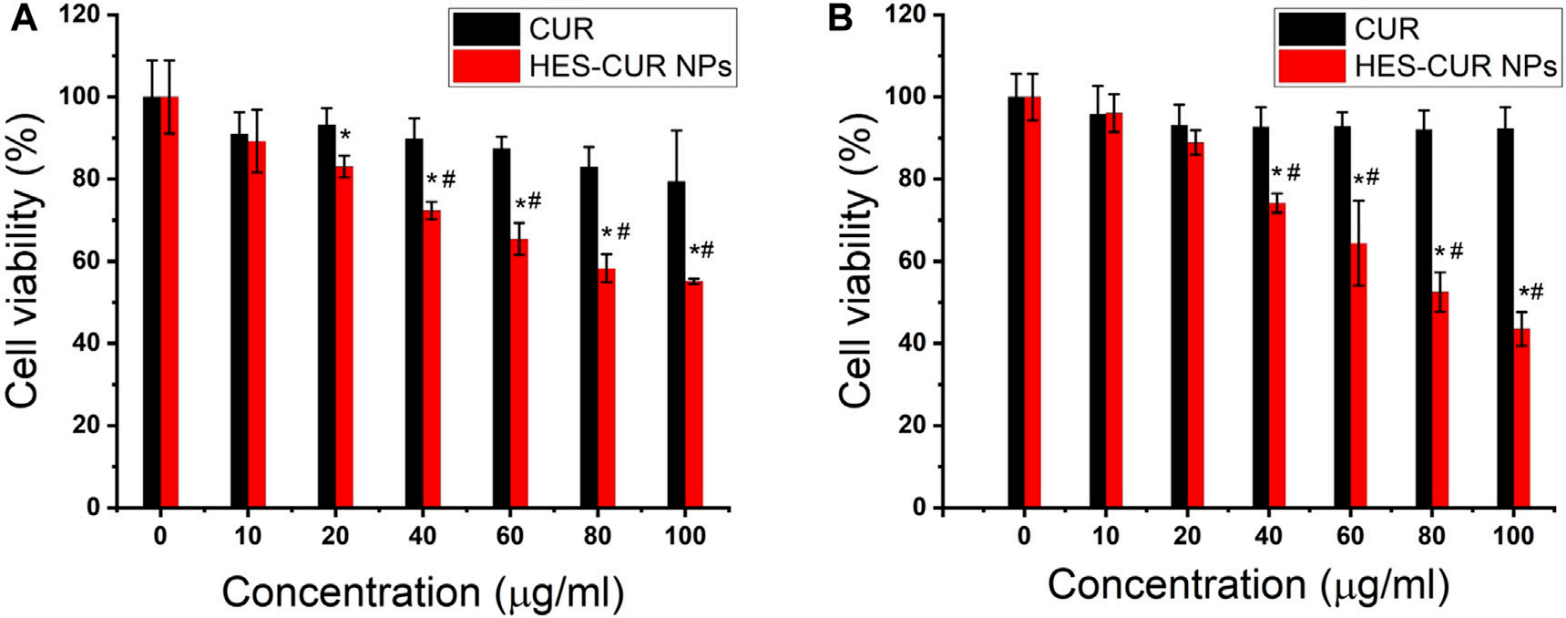
Effects of CUR and HES-CUR NPs on the viability of HepG2 cells incubated for 48 h. **(A)** CCK-8 assay and **(B)** cell counting. Data were expressed as mean ± SD, and the experiments were repeated thrice. *p < 0.05, compared to the control; ^#^ *p* < 0.05, comparisons of cell viability between CUR and HES-CUR NPs at the same concentration of CUR.

Figures 3A,B showed the antiproliferative effects of CUR and HES-CUR NPs in HepG2 cells. After 48 h of incubation, CUR itself had relatively lower antiproliferative effect in the HepG2 cells, and the cell viability was more than 80% even in the highest concentration of CUR (100 μg/ml). However, HES-CUR NPs had prominent antiproliferative effects, and the cell viability treated with the start of 20 μg/ml HES-CUR NPs was significantly lower when compared with the control group. And, this antiproliferative effect was significantly higher than that of CUR in HepG2 cells at the range of CUR concentrations (40–100 μg/ml). It should be noted that HES-CUR NPs (Supplementary Figure S1) were not toxic to 3T3 cells. Besides, many researchers proved that HES itself was not toxic as given by (Tang et al., 2019; Xu et al., 2020).

### *In vitro* Cellular Uptake

**FIGURE 4:**
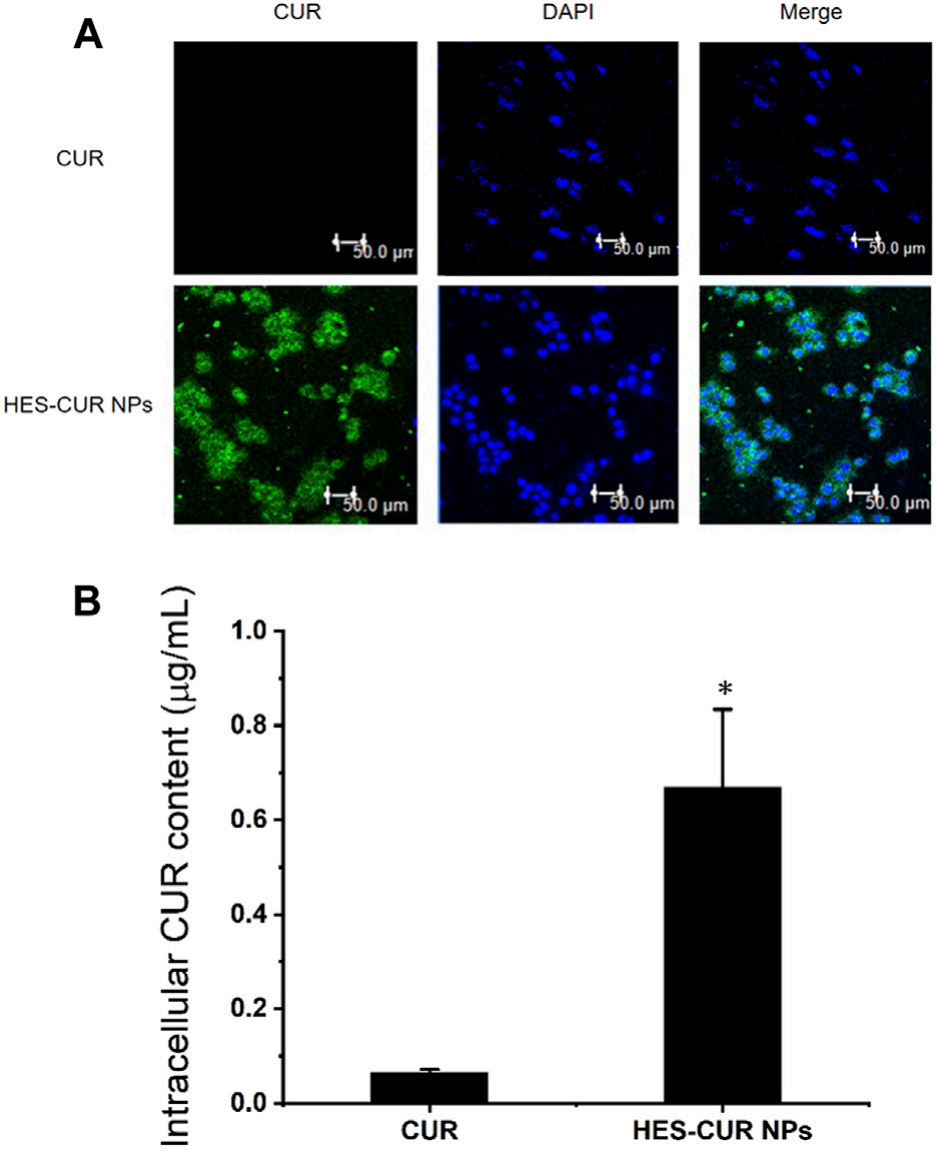
*In vitro* cellular uptake of CUR. **(A)** CLSM images of HepG2 cells incubated with CUR and HES-CUR NPs for 4 h, respectively. **(B)** Intracellular CUR content measured by HPLC. Data were expressed as mean ± SD, and the experiments were repeated thrice. *p < 0.05, compared to the CUR.

On the basic of intrinsic green fluorescence of CUR, we could clearly observe in Figure 4A that strong green fluorescence emerged in HES-CUR NP–treated HepG2 cells but not in CUR-treated cells. When the green fluorescence merged with the blue fluorescence area (the nucleus), it further indicated that HepG2 cells absorbed a mass of HES-CUR NPs, but less CUR was absorbed in the cells. Even when the HepG2 cell was treated with ultra-high concentration (375.00 μg/ml) of CUR, weak fluorescent signals were observed by CLSM (data not shown). The former result was confirmed by HPLC data, indicating that the mean intracellular content of CUR in the HES-CUR NP group (CUR treatment concentration: 125 μg/ml) was 0.67 ± 0.16 μg/ml, but the mean intracellular content of CUR in the CUR group (CUR treatment concentration: 375 μg/ml) was only 0.06 ± 0.005 μg/ml.

### Effects of the Curcumin and Hydroxyethyl Starch Curcumin NPs on HepG2 Cell Apoptosis

**FIGURE 5:**
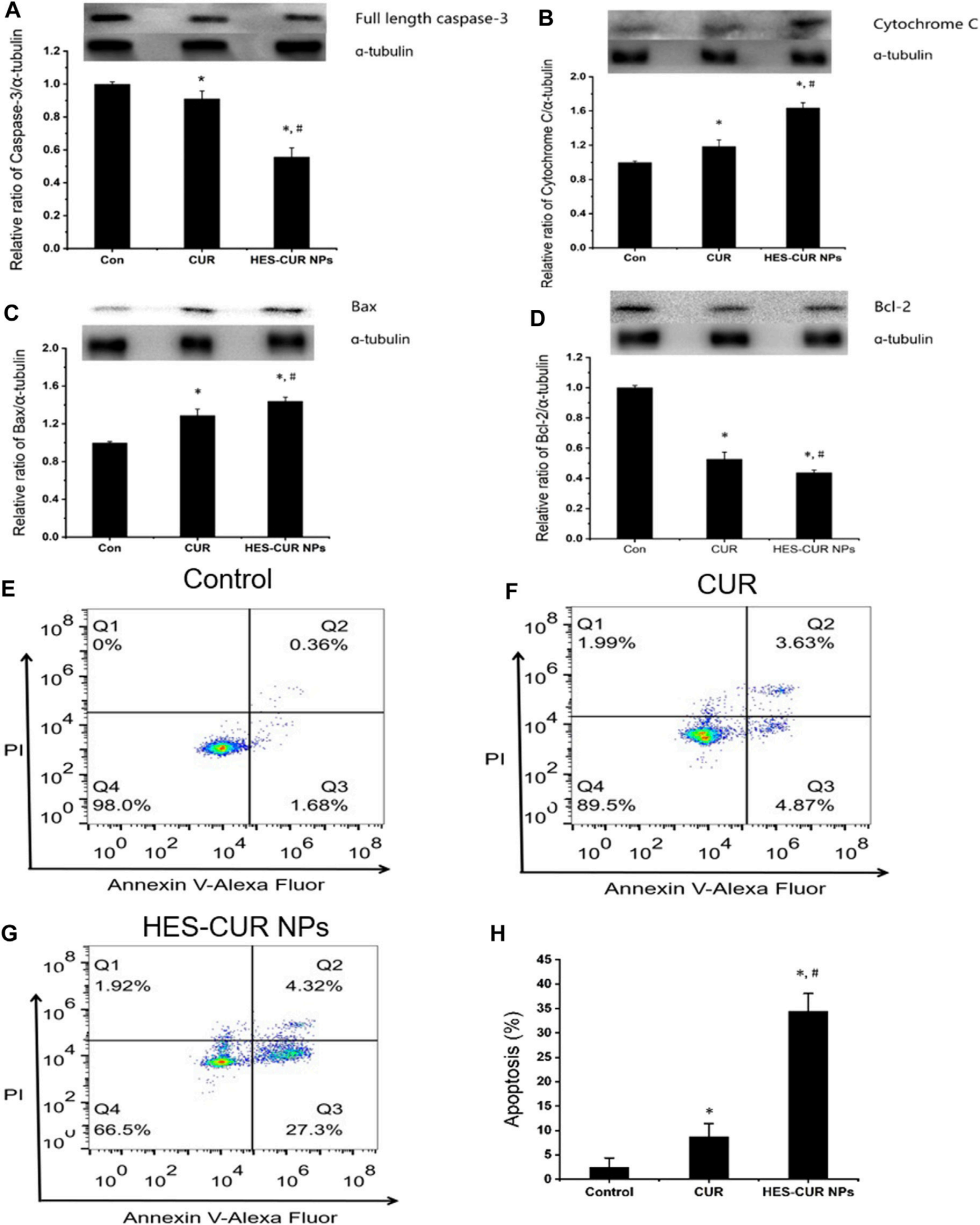
Effects of CUR and HES-CUR NPs on HepG2 cell apoptosis. **(A)** Full length caspase-3, **(B)** cytochrome c, **(C)** Bax, and **(D)** Bcl-2 expression levels were detected using Western blot. **(E–G)** Flow cytometric results of HepG2 cells after treating with CUR or HES-CUR NPs. Q4 = live cells, Q3 = early apoptosis, Q2 = late apoptosis, and Q1 = necrosis. **(H)** HepG2 cells were treated with CUR and HES-CUR NPs. Percentages of apoptotic cells were quantified by flow cytometry. Data were expressed as mean ± SD, and the experiments were repeated thrice. *p < 0.05 indicated significant differences from the control group; ^#^ *p* < 0.05 indicated significant differences from the CUR group.

The expression levels of apoptosis proteins, including full-length caspase-3, cytochrome c, Bax, and Bcl-2, after HepG2 cells being incubated with the CUR and HES-CUR NPs are shown in Figures 5A–D. First, relative to the control group, both HES-CUR NPs and CUR in HepG2 cells significantly induced lower expression levels of full-length caspase-3 and Bcl-2 (44 and 56.1%, respectively) and higher expression levels of cytochrome c and Bax. And the more significant changes of expression levels of four proteins were found in HES-CUR NP–treated HepG2 cells (HES-CUR NPs versus CUR: full-length caspase-3, 56 and 91% of the control; cytochrome c, 163 and 118% of the control; Bax, 144 and 129% of the control; and Bcl-2, 52 and 43% of the control).

Figures 5E–G showed the results of HepG2 cell apoptosis induced by CUR and HES-CUR NPs using flow cytometry. After HepG2 cells were incubated with CUR for 24 h, the proportions of early and late apoptosis were 4.87 and 3.63%, respectively. When HES-CUR NPs were used to treat HepG2 cells, the aforementioned proportions would rise to 27.3 and 4.32%, respectively. Thus, it was further proved that HepG2 cell apoptosis caused by HES-CUR NPs was significantly higher than that caused by CUR.

### Effects of Beclin-1 and LC3-II Level

**FIGURE 6:**
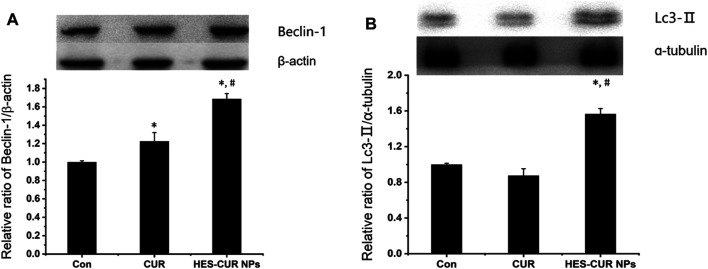
Effects of CUR and HES-CUR NPs on **(A)** Beclin-1 and **(B)** LC3-II expression levels. Data were expressed as mean ± SD, and the experiment was repeated thrice. *p < 0.05 indicated significant differences from the control group; ^#^ *p* < 0.05 indicated significant differences from the CUR group.

About the expression levels of Beclin-1 and LC3-II related to autophagy, relative to the control, HES-CUR NP–treated HepG2 cells had significantly higher levels of these two proteins (1.68 times and 1.56 times, respectively), but the expression of only Beclin-1 was increased by 1.22 times in CUR-treated HepG2 cells (Figure 6). More importantly, the observed results showed that HES-CUR NPs induced the expression levels of these two proteins far higher than CUR.

### Effects of Curcumin and Hydroxyethyl Starch Curcumin Nanoparticles on MMP and ATP Levels

**FIGURE 7:**
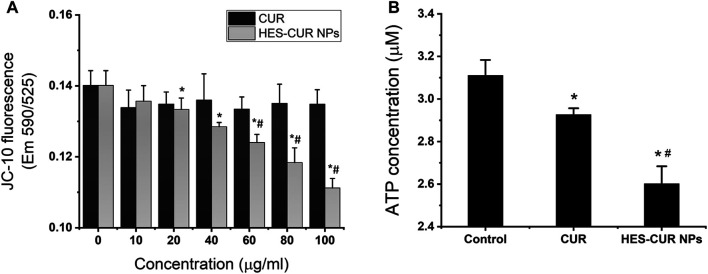
Effects of CUR and HES-CUR NPs on **(A)** MMP and **(B)** ATP levels. Data were expressed as mean ± SD, and the experiments were repeated thrice. *p < 0.05, compared to 0 ug/ml HES-CUR NPs; ^#^ *p* < 0.05, compared to the corresponding samples treated by CUR.

As shown in the results of the MMP in Figure 7A, HES-CUR NP–treated HepG2 cells displayed a concentration-dependent decrease of the fluorescence intensity but not in the CUR-treated group. We also found that the fluorescence intensity in HepG2 cells treated with the range of HES-CUR NP concentrations (20–100 μg/ml) was significantly lower than the control group. In addition, the decrease of fluorescence intensities in HES-CUR NP–treated HepG2 cells was more significant than that in the CUR-treated group at the range of CUR DLE (%) (60–100 μg/ml), indicating that HES-CUR NPs easily resulted in the depolarization of the MMP in HepG2 cells. It showed that HES-CUR NPs were more effective than CUR in causing decreased levels of MMP.

We further investigated the effects of CUR and HES-CUR NPs on ATP levels. The results displayed in Figure 7B showed that the ATP levels were significantly lower in both the CUR-treated and HES-CUR NP–treated groups than the control group. And the HES-CUR NPs causing the ATP shortage were more effective than the CUR itself (94 and 83.7% of the control).

## Discussion

### Difference of Antiproliferation Effect Between Curcumin and Hydroxyethyl Starch Curcumin Nanoparticles Dependent on Their Water Solubility

Although it is well known that CUR has great potential in the treatment of hepatic carcinoma, studies (Hu et al., 2020; Karimi et al., 2020; Karunagaran et al., 2005) have shown that the natural product CUR itself as an antitumor drug does not seem to be ideal, which is supported by our results showing that CUR had little inhibitory effects on the growth of HepG2 cells. The main reason for the low antitumor activity of CUR is its poor water solubility (Willenbacher et al., 2019). Therefore, the aim of this study was to improve the water solubility and stability of CUR to achieve better antitumor efficacy in HepG2 cells.

On the basis of our previous study (Chen et al., 2020), we successfully synthesized the soluble CUR-loaded nanoparticles by conjugating CUR to a food-derived biopolymer HES, and it was further confirmed by the characterization results of the ^1^H NMR spectrum, FT-IR spectrum, TEM, and DLS. In comparison with the report by Lee et al. (Lee et al., 2019), in which CUR liposomes were prepared by physical encapsulation, our HES-CUR NPs as lyophilized powders were more easily stored and showed better stability, thus it might avoid the burst release and sustain the release cycle. Besides, we further improved the DLE (%) of CUR, and the mean DLE (%) was about 25.61% and was almost two-fold higher than that of the previous study (Chen et al., 2020). When the HepG2 cells were treated by high loading of CUR, the HES-CUR NPs obviously exhibited the antitumor effect in a dose-dependent manner. It was closely associated with high uptake of CUR into the cells, which was observed by CLSM in this study.

In contrast, low antiproliferative activity was found in CUR-treated cells under the same concentration, mainly due to low amounts of CUR being absorbed in HepG2 cells. We also found that ultra-high concentrations of CUR were even used to treat HepG2 cells, but it could be seen that there was still little CUR in cells. HPLC results confirmed that intracellular content of CUR in the HES-CUR NP group was over ten times that of ultra-high concentration of the CUR-treated group. Remarkably, the high dosage of HES-CUR NPs could not result in the cytotoxic effect in normal 3T3 cells. It was mainly related to the CUR conjugated with HES via an acid–labile ester linker. Under such a condition, the acid-responsive HES-CUR NPs could be slowly released in normal cells (about 7.4 of pH) and rapidly released in tumor cells (about 5.0 of pH). Therefore, improved safety for normal tissues will make HES-CUR NPs of great potential in developing antitumor drugs.

### Difference of Antiproliferation Mechanism Between Hydroxyethyl Starch Curcumin Nanoparticles and Curcumin

Next, we explored the plausible mechanisms for the antiproliferation effect of HES-CUR NPs in HepG2 cells. Some studies (Moghtaderi et al., 2018; Ngai 2020; Yang et al., 2020) showed that CUR induced the apoptosis of many tumor cells, and it was basically consistent with the findings from this study, indicating that CUR increased the apoptosis rate of HepG2 cells as evidenced by the flow cytometric analysis. Moreover, we further found that HES-CUR NPs induced a nearly 5.6-fold increase of the cell apoptosis rate in comparison with CUR. It is well known that there are many pathways involved in apoptosis induction, including death receptors (extrinsic) and mitochondria (intrinsic) (Baig et al., 2016; Tomek et al., 2012).

In our study, HES-CUR NPs induced mitochondrial dysfunction and mitochondria-dependent HepG2 cell apoptosis with the following evidence. First, HES-CUR NPs could cause the significant reduction of MMP and facilitate ATP depletion, implying mitochondrial dysfunctions. Second, the impaired mitochondria induced the upregulation of proapoptotic Bax (Degterev et al., 2003) and downregulation of antiapoptosis Bcl-2 (Pistritto, Trisciuoglio, Ceci, Garufi and D'Orazi 2016) and prompted the cytochrome c release from mitochondria into the cytosol. Third, the upregulated Bax/Bcl-2 ratio and the increased release of cytochrome c further activated the executioner caspase-3 and ultimately resulted in the occurrence of mitochondria-mediated HepG2 cell apoptosis. Thus, it needs to be emphasized that HES-CUR NPs could directly increase the susceptibility of HepG2 cells to CUR, mainly due to the activation of the mitochondria-mediated intrinsic apoptosis pathway enhanced by HES-CUR NPs.

Notably, the upregulations of Beclin-1 and LC3-II levels were observed in HepG2 cells treated by HES-CUR NPs, but CUR-treated cells only had a higher expression level of Beclin-1. The data suggested that both HES-CUR NPs and CUR itself might induce autophagy, and HES-CUR NP–induced autophagy was more significant. The autophagic occurrence in this study was probably associated with the mitochondrial function. HES-CUR NPs resulting in mitochondrial dysfunction, such as ATP storage, could induce autophagy (Rabinowitz and White 2010). On the flip side, the low expression level of Bcl-2 protein caused by HES-CUR NPs could not only prevent the cell apoptosis by reducing the inhibition of cytochrome c (Pattingre et al., 2005) but also did not block the activation of autophagy by inhibiting the activity of Beclin-1 (Wu et al., 2010). So far, however, the question is still to be argued that autophagy may play a dual role in cancers where autophagy can either prevent tumorigenesis (Wang 2014) or promote tumor cell adaptation to stress (Majno and Joris 1995). So, further studies are required to clarify the different functions of autophagic mechanism and the importance of cross-regulation and balance between apoptosis and autophagy in different types of tumor cell response to specific drugs.

## Summary and Prospect

To sum up, HES-CUR NPs greatly improved the solubility, stability, bioavailability, and biocompatibility of CUR, thereby increasing the antiproliferative effect against HepG2 cells. The mechanistic investigations revealed that the enhanced antitumor activity was associated with the activation of mitochondria-mediated apoptosis and autophagy pathways. Hence, the data in this study indicated the potential of HES-CUR NPs to be developed as a promising therapeutic agent for the treatment of hepatocellular carcinoma, and also provided some insights into the mechanistic basis for the antitumor activity of HES-CUR NPs .

## Data Availability

The original contributions presented in the study are included in the article/Supplementary Material; further inquiries can be directed to the corresponding authors.
