# Supplementary material for: Hydroxyethyl Starch Curcumin Enhances Antiproliferative Effect of Curcumin Against HepG2 Cells via Apoptosis and Autophagy Induction
Source: Front Pharmacol. 2021 Nov 3;12:755054. doi: 10.3389/fphar.2021.755054 (PMC8595112; doi:10.3389/fphar.2021.755054)
Supplement: Supplementary file 1 [file DataSheet1.docx]

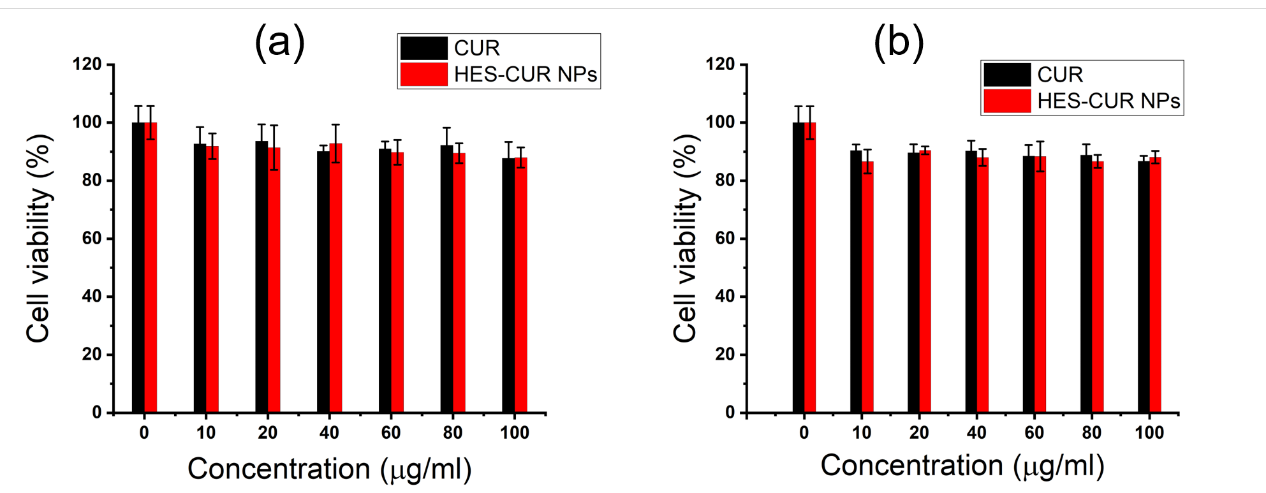


Supplementary Figure 1. Effects of CUR and HES-CUR on the viability of 3T3 cells incubated for 48 h. (a) CCK8 assay; (b) cell counting. Data were represented by mean ± SD, and the experiments were repeated three times.
